# Supplementary material for: Polarity-tunable magnetic tunnel junctions based on ferromagnetism at oxide heterointerfaces
Source: Nat Commun. 2015 Aug 13;6:8035. doi: 10.1038/ncomms9035 (PMC4557347; doi:10.1038/ncomms9035)
Supplement: Supplementary Information — Supplementary Figures 1-5, Supplementary Notes 1-4 and Supplementary References. [file ncomms9035-s1.pdf]

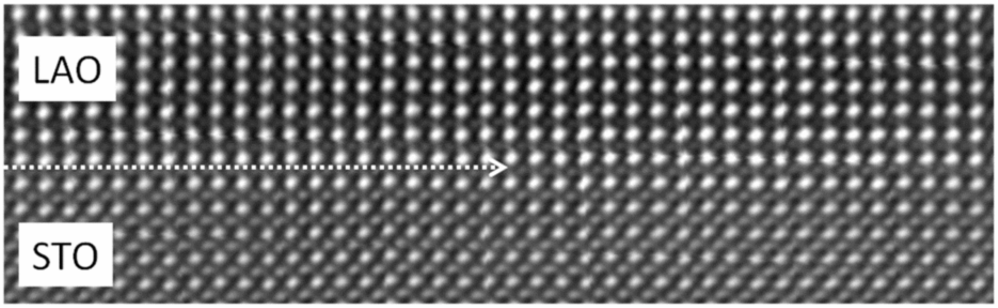

Supplementary Figure 1 | High-resolution transmission electron micrograph of the LAO/STO structure. LAO/STO interface indicated by the dotted line was atomically sharp and dislocation-free.

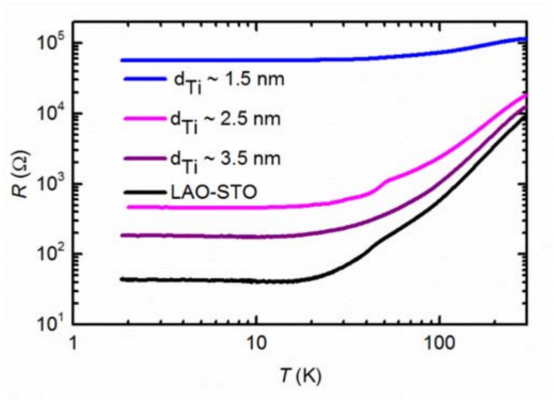

Supplementary Figure 2 | Control of the tunnelling resistance using the Ti interlayer thickness. Tunnelling resistance and LAO/STO sheet resistance as a function of temperature.

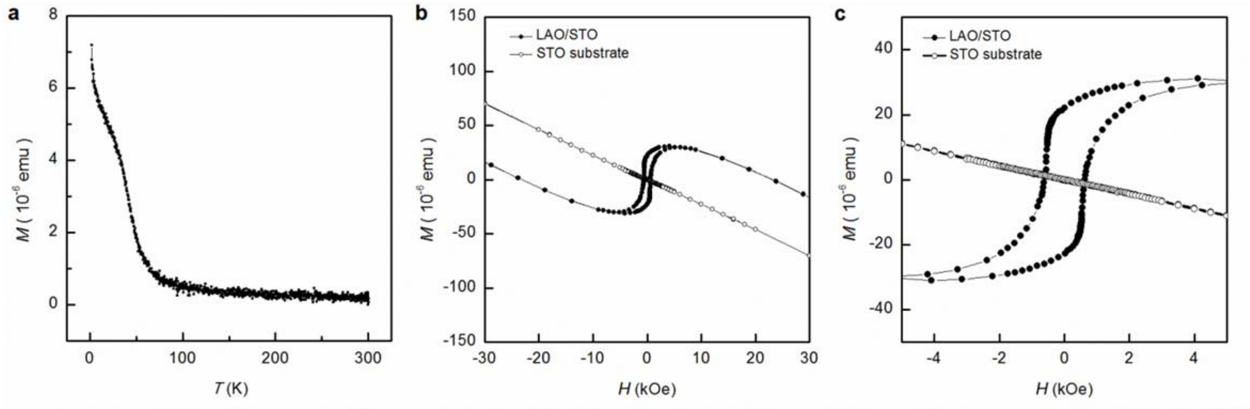

Supplementary Figure 3 | Magnetization measurement of the LAO/STO interface. **a**, Field cooling data as a function of temperature. A 7 kOe magnetic field is applied while the sample temperature increases from 2 to 300 K. **b**, Magnetization of the LAO/STO interface (solid circle) and bare STO substrate (open circle) versus the applied in-plane magnetic field measured at 2 K. **c**, Hysteresis ferromagnetic cycle of the LAO/STO interface.

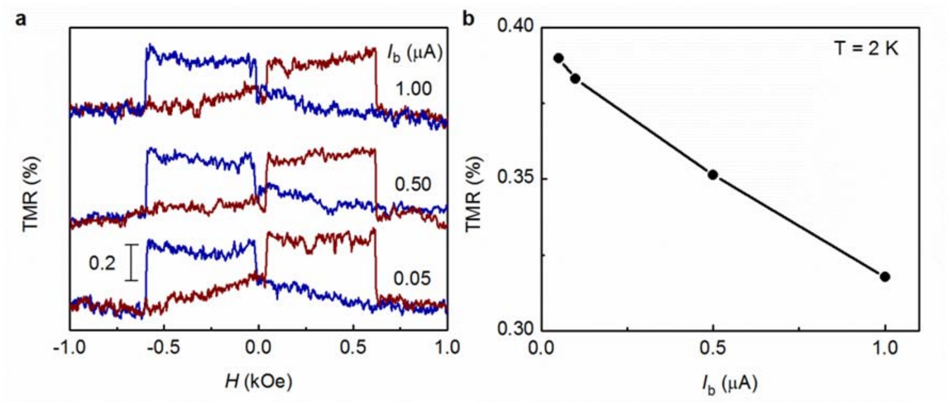

Supplementary Figure 4 | Bias current dependence of the magnetoresistance. **a**, The magneto-transport signals were acquired with various bias current varying from 50 nA up to 1 μA. **b**, TMR as a function of the bias current intensity.

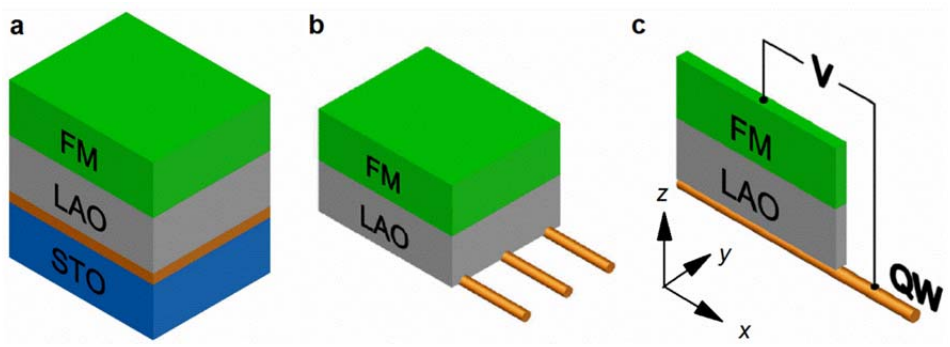

Supplementary Figure 5 | Theoretical model of the hybrid MTJs based on the LAO/STO heterostructure. **a**, Schematic of the device to measure the TMR between the LAO/STO interface and the ferromagnetic electrode (FM). **b**, Simplified model of the tunnel junction. The narrow conducting paths at the LAO/STO interface are regarded as quantum wires (QWs) with magnetic ordering and Rashba spin-orbit coupling. **c**, The further simplification of the model in **b**. For simplicity, the coupling between the parallel QWs is ignored and the tunnel junction is presented by a two-dimensional junction.

Supplementary Note 1: High-resolution transmission electron microscopy

Figure 1a in the main text shows a representative high-resolution (HR) TEM cross sectional view along the $[001]_{pc}$ direction (index pc - pseudocubic) and diffractograms taken by fast-Fourier transformation (FFT) of a $LaAlO_3$ (LAO) thin film grown on a $SrTiO_3$ (STO) substrate, with a thickness of approximately 3.69±0.05 nm (~10 unit cells) in Fig. 1a. The substrate causes a tensile strain in the LAO thin film because the bulk a-axis lattice constant of STO (a = 3.907 Å) is larger than that of LAO (a_{pc} = 3.792 Å). Our film is sufficiently thin, i.e. it can be expected to be elastically strained[1,2], that the difference in the lattice parameter along the in-plane direction is zero ($a_{STO} = a_{pc\text{-}LAO}$ = 3.907 Å) and the LAO c-axis lattice parameter ($c_{pc\text{-}LAO}$ = 3.692 Å) is very different from that of the STO. The interface between the LAO film and STO substrate is atomically flat and abrupt, and the STO thin film is free from extended defects (Supplementary Fig. 1). Strain relaxation in lattice-mismatched systems can normally be achieved through the introduction of misfit dislocations at the strained interface. However, based on the HRTEM micrograph and analysis of the diffractograms, we conclude that the LAO film is highly strained. In addition, it was confirmed that the titanium (Ti) contact layer was grown epitaxially on the LAO film, with a thickness of approximately 3.00±0.2 nm (Fig. 1a). From the diffractograms acquired by FFT, the crystallographic orientation relationship of $[5\bar{1}\bar{4}3]_{hcp\text{-}Ti}//[001]_{pc\text{-}LAO}$ and $(0\bar{1}11)_{hcp\text{-}Ti}//(020)_{pc\text{-}LAO}$ was identified between the Ti and LAO.

Supplementary Note 2: Ohmic contact formation

The electrical transport measurements were performed in a dc scheme. The forward bias (+V) was applied to the top metallic electrode. Fig. 1c displays the *V-I* characteristics of the tunnel junctions with various Ti layer thicknesses. A strong rectifying behaviour was clearly observed in the Co/LAO/STO device, indicating that a Schottky barrier was formed at the interface between the ferromagnetic metal and oxide structure[3,4]. The tunneling conductance increased a few orders of magnitude when a thin nonmagnetic metal layer was inserted between Co and LAO. The Ohmic contact was roughly formed in the d_{Ti} ~ 2.5 nm case, which was confirmed by the symmetry of the *V-I* curve. When a 2.5 nm Ti layer was inserted, the junction resistance was a few times higher than the sheet resistance of the LAO/STO interface (Supplementary Fig. 2) and the contact resistance-area (RA) product varied from 1.25 to 2.50 Ωcm^2.

Supplementary Note 3: Magnetization measurement

The magnetism of the LAO/STO heterointerface was measured using a Quantum Design magnetic property measurement system (MPMS). Supplementary Fig. 3a shows the field-cooled magnetization as a function of temperature. An in-plane magnetic field of 7 kOe was applied along one of the oxide crystallographic axes while the temperature increased from 2 to 300 K. The positive values below 100 K reveal the paramagnetic properties of the oxide sample. Furthermore, the magnetic hysteresis cycle indicates that a ferromagnetic loop is embedded in a large diamagnetic response to the magnetic field sweeping from – 70 to 70 kOe, while the bare STO substrate exhibits only the diamagnetic response (Supplementary Fig. 3b). Supplementary Fig. 3c presents the ferromagnetic data subtracted from the diamagnetic background. The

coercive force of the LAO/STO interface was determined at ~600 Oe, which is consistent with that reported in ref. 5.

Supplementary Note 4: Bias-current dependence of the tunneling signals

The magneto-transport measurement was conducted while various bias currents were applied to the hybrid magnetic tunnel junctions (MTJs). As observed in Supplementary Fig. 4, the tunneling magnetoresistance (TMR) slightly depends on the bias current, which suggests that the Schottky barrier between the metallic electrode and LAO thin film is sufficiently reduced. Therefore, the direct spin injection into the LAO/STO interface is enabled. As the electrical current increases, the small reduce of the TMR signals is merely due to the scattering between spin-polarized electrons, which causes the loss of spin polarization. However, as a higher bias current (~ several tens of micro Amperes) was applied, TMR signal was completely suppressed. This strong bias dependence is in contrast with the ordinary giant magnetoresistance (GMR) behavior, which is insensitive to the bias current.

Supplementary References

1. Bark, C. W. *et al*. Tailoring a two-dimensional electron gas at the $LaAlO_3/SrTiO_3$ (001) interface by epitaxial strain. *Proc. Natl. Acad. Sci*. **108**, 4720-4724 (2011).

2. Pauli, S. A. *et al*. Evolution of the interface structure of $LaAlO_3$ on $SrTiO_3$. *Phys. Rev. Lett.* **106**, 036101 (2011).
3. Singh-Bhalla, G. *et al.* Built-in and induced polarization across $LaAlO_3/SrTiO_3$ heterojunctions. *Nature Phys.* **7**, 80-86 (2010).
4. Reyren, N. *et al.* Gate-controlled spin injection at $LaAlO_3/SrTiO_3$ interfaces. *Phys. Rev. Lett.* **108**, 186802 (2012).
5. Ariando *et al*. Electronic phase separation at the $LaAlO_3/SrTiO_3$ interface. *Nature Commun*. **2**, 188-194 (2011).
